# Effects of Different Training Intensity Distributions on Endurance Capacity in Breast and Prostate Cancer Survivors: A Randomized Controlled Trial

**DOI:** 10.1002/ejsc.12287

**Published:** 2025-04-08

**Authors:** Nikolai Bauer, Justine Schneider, Kathrin Schlüter, Joachim Wiskemann, Friederike Rosenberger

**Affiliations:** ^1^ Working Group Exercise Oncology Department of Medical Oncology National Center for Tumor Diseases (NCT) Heidelberg University Hospital Heidelberg Germany; ^2^ Division of Health Sciences German University of Applied Sciences for Prevention and Health Management Saarbruecken Germany

**Keywords:** aerobic fitness, chronic disease, endurance, health

## Abstract

This study aimed to compare the effects of isocaloric polarized and threshold training intensity distribution on endurance capacity in breast and prostate cancer survivors. A total of 28 breast and 27 prostate cancer survivors were randomly assigned to a polarized (POL, *n* = 27 (13 women), age 60 ± 8 years, peak oxygen uptake (VO_2peak_) 23 mL·min^−1^ kg^−1^), or threshold training group (ThT, *n* = 28 (15 women), age 59 ± 10 years, VO_2peak_ 23 mL·min^−1^ kg^−1^) who completed two sessions per week on a cycle ergometer over 12 weeks. Exercise duration was adapted to obtain equivalent energy expenditure in both groups. Cardiopulmonary exercise and verification tests were performed to determine endurance capacity (VO_2peak_, peak power output (PPO), ventilatory threshold (VT_1_), blood lactate thresholds (LT_1_ and IAT)), and maximal exhaustion. POL did not achieve the planned polarized intensity distribution and rather performed a pyramidal training. Pyramidal and threshold training significantly (*p* < 0.001) improved endurance capacity regarding VO_2peak_ (0.09 and 0.12 L·min^−1^), PPO (27 and 17W), power output at VT_1_ (11 and 13W), oxygen uptake at VT_1_ (0.09 and 0.11 L·min^−1^), power output at LT_1_ (7 and 12W), and power output at IAT (12 and 14W). No difference was found between groups, but ThT required significantly (*p* < 0.001) less time than pyramidal training to achieve the described improvements (59 ± 1 min/week vs. 76 ± 11 min/week). Comparison of isocaloric training intensity distributions revealed no significant differences between groups (Pyramidal: 170 ± 43 kJ/session, ThT: 175 ± 35 kJ/session, *p* = 0.10). Pyramidal and isocaloric threshold training resulted in comparable effects on endurance capacity in cancer survivors, with ThT requiring significantly less time for these effects.

**Summary**

- This is the first study to compare two different isocaloric training intensity distributions regarding their effects on endurance capacity in cancer survivors.
- Planned polarized could not be achieved and participants rather conducted a pyramidal intensity distribution.
- Pyramidal and isocaloric threshold training appear to be safe and feasible for breast and prostate cancer survivors resulting in similar significant improvements in endurance capacity, although threshold training was more time efficient.

## 1 Introduction

Breast and prostate cancer are among the most common types of cancer worldwide and consequently major causes of cancer‐related mortality (Sung et al. 2021). However, an increasing number of breast and prostate cancer patients survives due to new individualized therapy options (e.g., neoadjuvant and adjuvant chemotherapy) (Siegel et al. 2023). Unfortunately, cancer survivors often suffer from various side effects of cancer and its treatment such as reduced physical performance or fatigue (Bower 2014; Jones et al. 2012). In this context, structured physical exercise is explicitly recommended since numerous studies have demonstrated the positive effects of structured physical exercise on biopsychosocial parameters such as cardiorespiratory and neuromuscular fitness or psychological health (e.g., health‐related quality of life) (Campbell et al. 2019; Martínez‐Vizcaíno et al. 2023). Especially the positive effects on cardiorespiratory fitness are of immense clinical importance since higher cardiorespiratory fitness is associated with lower overall cancer incidence and mortality (Kunutsor et al. 2024).

However, it is necessary to find the most adequate and effective training regimen to maximize the benefits on cardiorespiratory fitness and to avoid overtraining (Buffart et al. 2017; Hayes et al. 2019). In order to do so, the distribution of training intensity is essential (Seiler and Kjerland 2006). Training intensity distribution has been examined several times in elite endurance athletes. Thus, various studies (Filipas et al. 2022; Stöggl and Sperlich 2014) compared different distributions of training intensity with regard to their effects and were able to show that a so‐called polarized training intensity distribution can positively influence endurance performance. A polarized training intensity distribution consists of the following intensity distribution: 75%–80% low‐intensity, 5% threshold intensity, and 15%–20% high‐intensity (Treff et al. 2019). Moreover, retrospective analyses showed that endurance athletes often use a polarized training intensity distribution for training (Billat et al. 2001; Seiler and Kjerland 2006). Such retrospective analyses provide first information about the most effective training regimens also for cancer patients, although they cannot replace intervention studies. Foster et al. (2022) concluded that the polarized distribution of training intensity is “optimal” for endurance athletes. Since then, there has been a lively discussion about polarized training and its effects but there is still no consensus. Burnley et al. (2022) relativized the superiority of polarized training over other training intensity distributions postulated by Foster et al. (2022) and emphasized the positive effects of pyramidal training on endurance capacity. Altogether, further investigations are needed in the context of polarized and other training intensity distributions.

The current literature (Buffart et al. 2018; Herranz‐Gómez et al. 2022; Neuendorf et al. 2023; Scott et al. 2018) shows that in recent years, a rising number of studies has investigated the effects of different training intensity distributions such as high‐intensity interval training (HIIT) or threshold training in exercise oncology. Those studies indicate that cancer patients and survivors are able to perform endurance training at low‐intensity (Zone 1), threshold intensity (Zone 2), and high‐intensity (Zone 3) according to the triphasic model (Skinner and Mclellan 1980). In addition, the studies revealed that low, moderate, and high‐intensity training can be considered safe for breast and prostate cancer patients after the end of primary therapy (i.e., surgery and/or radio therapy and/or chemotherapy) and can lead to increased cardiovascular and physical fitness, as well as mental well‐being. However, despite the recent popularity of polarized training intensity distribution in elite sports, to the best of our knowledge, no study exists that examined the effects of polarized training intensity distribution in cancer survivors or compared it with isocaloric threshold training.

For this reason, we investigated the effects of polarized training in comparison with isocaloric threshold training in breast and prostate cancer survivors after the end of primary therapy. To do so, we adapted a polarized training regimen to the capabilities of cancer survivors. Based on the aforementioned studies, we hypothesized that polarized training is superior to threshold training in terms of endurance capacity.

## 2 Materials and Methods

### 2.1 Study Design

A single‐center randomized controlled clinical trial (TOP‐Study, clinicaltrials. gov: NCT02883699) involving a four‐arm exercise intervention (two aerobic exercise groups and two resistance exercise groups) was performed. The results of the two aerobic exercise groups (primary outcome results) are reported here, as the four‐arm design was chosen to compare secondary endpoints (patient reported outcomes) between all groups. Randomization was based on a minimization procedure for i) age; ii) cancer entities (also corresponding to sex); iii) current hormone treatment; and iv) fitness level (characterized by relative peak oxygen uptake (VO_2peak_) in the aerobic exercise groups). The intervention included two exercise sessions per week performed over a 12‐week period resulting in a total of 24 exercise sessions. The two aerobic training groups were designed as follows: The participants in the “threshold training group” (ThT) performed continuous training at vigorous intensity. Participants in the “polarized training group" (POL) performed polarized training with equal energy expenditure as ThT. Respectively, the intensity varied between HIIT and low‐intensity continuous training (LICT) (Faude et al. 2008). An assessment was conducted before and after 12 weeks of the intervention. The study was in accordance with the standards of the Declaration of Helsinki and approved by the ethics committee of the Medical Faculty of Heidelberg (S‐347/2016). Additionally, written informed consent was obtained from all participants.

### 2.2 Participants

All participants were recruited at the National Center for Tumor Diseases (NCT) Heidelberg, Germany or from local oncologists as well as via a cancer registry and advertisement in self‐help magazines. Participants had to fulfill the following inclusion and exclusion criteria to be eligible for the study: i) diagnosed with nonmetastatic (M0) breast cancer or nonmetastatic or metastatic prostate cancer (M0 or M1, except for bone or brain metastases and with PSA evidence of stable disease); ii) six to 52 weeks after the end of primary therapy (i.e., surgery and/or radio therapy and/or chemotherapy); iii) 18–75 years of age; iv) no regular endurance or resistance training (i.e., > 1 session/week) since diagnosis or within the last 6 months; v) no diagnosis with additional other type of cancer; and vi) no severe comorbidities that precluded participation in exercise testing or training (e.g., acute infectious diseases, severe cardiorespiratory, renal or neurological diseases).

### 2.3 Outcome Measures

The primary endpoint was VO_2peak_. This and secondary endpoints regarding endurance capacity (peak power output (PPO), power output at VT_1_ (PO_VT1_), oxygen uptake at VT_1_ (V0_2_ at VT_1_), power output at LT_1_ (PO_LT1_) and power output at IAT (PO_IAT_) as well as indicators of maximal exhaustion (peak heart rate (HR_peak_), peak blood lactate concentration (bLA_peak_), and peak respiratory exchange ratio (RER_peak_)) were determined by two independent investigators during a cardiopulmonary exercise test (CPET) with a subsequent verification test on an electronically braked cycle ergometer (Ergoselect 100 P; Ergoline, Bitz, Germany).

#### 2.3.1 CPET and Verification Test Protocol

Specifically, the CPET started at 20 W and the work rate increased by 10 W every minute until exhaustion. Participants had to maintain a cadence of 60–80 rpm and exert maximum efforts. The verification test was performed 10 min after the end of CPET according to the protocol of Schneider, Schlüter, Sprave, et al. (2020a) to verify the achievement of VO_2peak_. Gas exchange was measured using a breath‐by‐breath gas analysis system (Ergostik; Geratherm Respiratory, Bad Kissingen, Germany). Validity of the Ergostik (±3% or 50 mL/s) was confirmed by Van Hooren et al. (2024). HR was obtained from a 12‐lead electrocardiogram (ECG) (CardioPart 12 Blue; Amedtec, Aue, Germany) and blood pressure was measured manually and monitored every 2 min. RPE (using 6 to 20 Borg scale) was recorded. bLA_peak_ was measured from blood samples taken at the end of exercise and in the first, third, and fifth min of the recovery period from the hyperemized (Finalgon balm) earlobe using 10‐μL end‐to‐end capillaries. Blood samples were analyzed with an enzymatic–amperometric method (Super GL compact; Hitado, Möhnesee, Germany).

#### 2.3.2 Assessment of Outcome Measures

VO_2peak_, RER_peak,_ and HR_peak_ were considered the highest 20 s average values during or immediately after CPET. VO_2peak_ of the verification test was considered the highest 20 s average value during the verification test. Ventilatory threshold 1 (VT1) was determined by two experienced investigators. Lactate threshold (LT, minimum lactate equivalent) and individual anaerobic lactate threshold (IAT, 1 mmol/L above minimum lactate equivalent) were determined to derive intensity prescriptions (Software Ergonizer, Freiburg, Germany). Power output at VT_1_ (PO_VT1_), power output at LT_1_ (PO_LT1_), PO_IAT_ and PPO were recorded.

#### 2.3.3 Assessment of Maximal Exhaustion

The CPET was considered maximal if a minimum of two of the following criteria were fulfilled: i) ≤ 3% difference between VO_2peak_ at the CPET and VO_2peak_ at the verification test; ii) RER_peak_ ≥ 1.1; iii) bLA_peak_ ≥ 8 mmol·L‐1; and iv) HR_peak_ ≥ 200 bpm minus age (Ferguson 2014; Midgley et al. 2007; Schneider, Schlüter, Wiskemann, et al. 2020b). Participants who did not reach maximal exhaustion were not excluded from the analysis, as is common in the field of exercise oncology, to avoid that this study lacks comparability to other studies in the field. Therefore, the main purpose of the analysis of maximal exhaustion was to investigate differences between groups and measurement time points.

### 2.4 Training Intensity Distribution

For both groups, training intensity was specified by HR and categorized using the triphasic model according to Skinner and Mclellan (1980): i) Zone 1: heart rate below LT_1_; ii) Zone 2: heart rate between LT_1_ and LT_2_; and iii) Zone 3: heart rate above LT_2_. Thus, training intensity distribution was quantified using the heart rate time‐in‐zone method and additionally the polarization index (Treff et al. 2019) was calculated to determine the training intensity distribution in detail. In this context, training intensity distributions with values > 2.00 were defined as polarized and values ≤ 2.00 as nonpolarized.

### 2.5 Intervention

All sessions were supervised by trained exercise professionals and occurred in an indoor gym on a cycle ergometer. Participants of the ThT performed two sessions per week of vigorous intensity continuous training slightly below IAT (approximately 97% of IAT) lasting 30 min each. POL also conducted two sessions per week. However, to achieve a polarized training intensity distribution, one session per week was designed as HIIT and the other as LICT. HIIT lasted 38 min per session and started with a 10‐min warm‐up at 70% HR_peak,_ followed by 4 × 4 min intervals at 85%–95% HR_peak_, which were interspersed with 3 min recovery phases at 70% HR_peak_ and finished with a 3‐min cooldown (70% HR_peak_) (Schneider, Schlüter, Sprave, et al. 2020a). LICT was conducted at the LT and the duration of the sessions was individually calculated to ensure that the two aerobic training groups trained isocaloric. The isocaloric training design was used to ensure that differences in training effects are attributable to the intensity distribution and not to total training load or energy expenditure, respectively. Work rates were increased/decreased if the HR decreased/increased below or over the target zone to maintain adequate intensity throughout the intervention. For monitoring the HR, a telemetric system (Polar Electro, Kempele, Finland) was used. No additional, specifically designed motivational program was implemented, but the exercise professionals provided the participants with regular feedback to keep their motivation high. Possible adverse events, premature session terminations, and training compliance were documented. Moreover, energy expenditure in kilojoules per session and week was calculated based on the power output of the participants in the training sessions.

### 2.6 Statistical Analysis

An appropriate sample size was estimated a priori based on VO_2peak_ with a minimum worthwhile difference in VO_2peak_ between groups of 10%, a within‐subject variation in VO_2peak_ of 5.6% (Katch et al. 1982), *α* = 0.05, and power = 80%. The estimation yielded *n* = 20 assessable participants per group. At least 30 participants per group were included to account for possible dropouts. All statistical analyses were performed using JASP software program (Version 0.16.2; JASP Team, Amsterdam, the Netherlands). Means and standard deviations (SD) were calculated for all data. Normal distribution was verified with the Shapiro–Wilk test and visual inspection, and homogeneity of variance was assessed with the Levene test. Normal distribution and equal variances were present for all parameters. An independent samples *t*‐test and the chi‐squared test were used to compare groups for differences in baseline data. To compare the changes in the analyzed parameters over the intervention period between groups, a mixed ANOVA with within‐subject factor time (2, baseline testing—post‐intervention testing) and between‐subject factor group (2, ThT, POL) was used with an alpha level of *p* < 0.05. In addition, the effect size (ES) eta‐squared (η^2^) between pre and post‐testing was calculated for all variables (0.01 (small ES), 0.06 (medium ES), and 0.14 (large ES)) (Cohen 1988). For the comparison of the energy expenditure and the training intensity distribution of both groups, the independent samples *t*‐test was used.

## 3 Results

**FIGURE 1:**
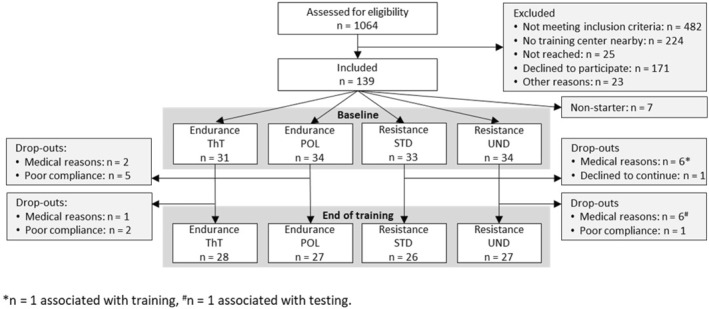
Participant flow chart.

A total of 65 cancer survivors (35 breast cancer and 30 prostate cancer) were randomized to the aerobic exercise groups after the baseline assessment. Finally, 28 participants of the ThT (15 breast cancer and 13 prostate cancer) and 27 participants of the POL (13 breast cancer and 14 prostate cancer) completed post‐intervention testing and could be included in the analysis. Three participants dropped out over the intervention period due to medical reasons and seven participants due to a lack of compliance. Consequently, group‐specific dropout rates of 20.6% for POL and 9.7% for ThT were computed. Figure 1 shows the participant flowchart in detail. The anthropometric and medical characteristics of the participants are presented in Table 1. There were no baseline differences between the groups regarding all examined parameters.

**TABLE 1:** Participants' characteristics.

	Training	
	ThT	POL
Type of cancer, *n* (%)	
Breast cancer	15 (53.6)	13 (48.1)
Prostate cancer	13 (46.4)	14 (51.9)
Age [years] (mean ± SD)	59 ± 10	60 ± 8
Height [cm] (mean ± SD)	173 ± 8	170 ± 8
Weight [kg] (mean ± SD)	82 ± 19	80 ± 19
BMI [kg/m^2^] (mean ± SD)	27.5 ± 5.5	27.4 ± 5.0
VO_2peak_ [L/min] (mean ± SD)	1.86 ± 0.39	1.83 ± 0.46
VO_2peak_ [mL/min/kg] (mean ± SD)	23.00 ± 4.49	23.10 ± 4.04
Time since diagnosis [months] (mean ± SD)	14 ± 7	21 ± 18
Time since end of therapy [wk] (mean ± SD)	25 ± 17	26 ± 12
Tumor staging, *n* (%)	
0	0 (0)	1 (3.7)
I	11 (39.3)	9 (33.3)
II	6 (21.4)	9 (33.3)
III	6 (21.4)	6 (22.2)
IV	1 (3.6)	0 (0)
Unclear	4 (14.3)	2 (7.4)
Cancer treatment, *n* (%)	
Surgery alone	5 (17.9)	2 (7.4)
Radio therapy alone	1 (3.6)	4 (14.8)
Surgery + radio therapy	12 (42.9)	14 (51.9)
Surgery + chemotherapy	1 (3.6)	1 (3.7)
Surgery + radio therapy + chemotherapy	9 (32.1)	5 (18.5)
Others	0 (0)	1 (3.7)
Additional hormone therapy	16 (57.1)	16 (59.3)
Additional antibody therapy	5 (17.9)	2 (7.4)

No adverse events occurred during training regimens or related testing. Participants of ThT performed an average of 23.96 ± 0.19 sessions, corresponding to a compliance of 99.85%. Only one session was terminated prematurely because of schedule conflicts. Thus, the participants of the ThT performed 712 ± 17 min of vigorous cycling or 59 ± 1 min/week. POL performed 23.96 ± 0.19 sessions. This equals a compliance of 99.85%, and only one session was discontinued due to an asthma attack. Participants of POL engaged in a total of 907 ± 134 min (HIIT: 453 ± 8 min (49.96%); LICT: 454 ± 136 min (50.04%)) or 76 ± 11 min/week, resulting in significant differences (*p* < 0.001) between the groups with respect to total training time.

### 3.1 Training Intensity Distribution

**FIGURE 2:**
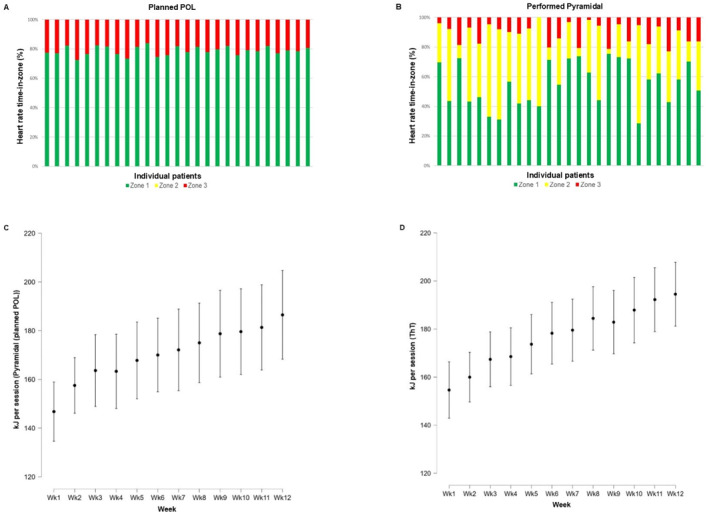
(A and B) Individual training intensity distributions for planned POL and performed pyramidal training. (C and D) Energy expenditure per group and sessions for the performed pyramidal training (planned POL) and ThT.

The performed training time in Zone 1 (*p* < 0.001), Zone 2 (*p* < 0.05), and Zone 3 (*p* < 0.05) differed between POL and ThT (Supplementary Material). In addition, planned and performed training intensity distributions differed noticeably for most participants of POL (Figure 2A and 2B), resulting in an average intensity distribution of the planned polarized training group corresponding to a pyramidal training intensity distribution. In the following, we will therefore compare the performed pyramidal intensity distribution with the intensity distribution of the threshold training.

### 3.2 Endurance Capacity

Changes in endurance capacity did not differ between groups (Table 2 and Supplementary Material). However, VO_2peak_ (*p* < 0.001), PPO (*p* < 0.001), PO_VT1_ (*p* < 0.001), VO_2_ at VT_1_ (*p* < 0.001), PO_LT1_ (*p* < 0.001), and PO_IAT_ (*p* < 0.001) improved significantly over time within both groups.

**TABLE 2:** Changes in endurance capacity.

	Pyramidal training group (planned POL) (mean ± SD)		Threshold training group (mean ± SD)		ANOVA *p*		
	PRE	POST	PRE	POST	Time (η^2^)	Group (η^2^)	Group × time (η^2^)
VO_2peak_ (L·min^−1^)	1.83 ± 0.46	1.92 ± 0.47	1.86 ± 0.39	1.98 ± 0.47	< 0.001^a^ (0.01)	0.71 (0.002)	0.62 (< 0.001)
PPO (W)	139 ± 32	166 ± 69	139 ± 29	156 ± 28	< 0.001^a^ (0.06)	0.58 (0.004)	0.37 (0.003)
PO_VT1_ (W)	52 ± 14	63 ± 17	55 ± 15	68 ± 21	< 0.001^a^ (0.11)	0.34 (0.01)	0.73 (< 0.001)
VO_2_ at VT_1_ (L·min^−1^)	0.93 ± 0.19	1.05 ± 0.21	1.01 ± 0.22	1.12 ± 0.26	< 0.001^a^ (0.06)	0.23 (0.02)	0.79 (< 0.001)
PO_LT1_ (W)	63 ± 14	70 ± 15	62 ± 12	74 ± 16	< 0.001^a^ (0.10)	0.58 (0.004)	0.06 (0.008)
PO_IAT_ (W)	88 ± 17	100 ± 20	90 ± 17	104 ± 22	< 0.001^a^ (0.10)	0.52 (0.006)	0.41 (0.001)

^a^ Significant for *p* < .05.

### 3.3 Energy Expenditure

The training energy expenditure of both groups did not differ significantly (Pyramidal: 170 ± 43 kJ/session, ThT: 175 ± 35 kJ/session, *p* = 0.10). As demonstrated in Figures 2C and 2D, both groups had a similar increase in energy expenditure over time (Pyramidal: 3 ± 13 kJ/session; ThT: 4 ± 13 kJ/session).

### 3.4 Criteria for Maximal Exhaustion

Ninety‐three percent and 85% of the pyramidal training group as well as 93% and 93% of ThT fulfilled at least 2 criteria regarding maximal exhaustion at baseline and post‐intervention testing, respectively. In this context, 66% (Pyramidal = 56%, ThT = 77%) and 75% (Pyramidal = 67%, ThT = 82%) of the participants met the verification criterion at baseline and post‐intervention assessments. There were no group or interaction effects with respect to parameters regarding maximal exhaustion (supplementary material). HR_peak_ (*p* = 0.19), RER_peak_ (*p* = 0.17), and RPE (*p* = 0.51) did not change over time in either group. In contrast, bLA_peak_ increased significantly from baseline to post‐intervention assessments in both groups (*p* < 0.05).

## 4 Discussion

The key findings of this study are that (i) most participants of the planned polarized training group rather performed a pyramidal training intensity distribution. (ii) Performed pyramidal and threshold training with isocaloric energy expenditure led to significant improvements in endurance capacity with similar improvements in both groups. (iii) Participants of ThT required significantly less training time (−17 min/week) to improve endurance capacity than the pyramidal training group. (iv) No training‐related adverse events occurred due to one of the two training regimens. Thus, our hypothesis that polarized training is superior to threshold training in terms of endurance capacity could not be clearly verified as we did not achieve a polarized intensity distribution.

The present study indicates that most of the analyzed breast and prostate cancer survivors were not able to perform a planned polarized intensity distribution (75%–80% Zone 1, 5% Zone 2, and 15%–20% Zone 3), but rather conducted a pyramidal intensity distribution with Zone 1 > Zone 2 > Zone 3. There are several possible reasons for this surprising result. First, patients in this study were unable to follow the high intensities as part of polarized training over the intervention period, which is indicated by the slightly reduced training volume in Zone 3 (11%). This is supported by the findings of Nilsen et al. (2018) investigating specific methods regarding the report of training adherence. The authors showed that although training at high intensity was feasible for most of the prostate cancer patients investigated after curative‐intent radical prostatectomy, consideration of the individual data also pointed to the challenges of high‐intensity training and resulting dose adjustments. Second, our participants showed a reduced cardiorespiratory fitness compared to healthy individuals (Pescatello et al. 2014). Such a reduced cardiorespiratory fitness could have been the reason for a slower decrease in the participants' heart rate during the recovery phases of HIIT sessions leading to less time in Zone 1. Moreover, the use of the heart rate for the quantification of the training intensity distribution was potentially another reason for the difference between planned and completed intensity distributions. The time‐delayed response of the heart rate might have influenced the observed intensity distribution (Sperlich et al. 2023) and led to a lower time spent in Zone 1 and Zone 3. With regard to those aspects, the consideration of training intensity distribution could be a further tool for an adequate reporting of training adherence in exercise oncology. However, avoiding a delayed heart rate response using session RPE to quantify the training intensity distribution might be beneficial in exercise oncology.

Despite the important difference between planned and completed intensity distributions, there are also various reasons for the lack of difference between the two training groups. Considering the intensity distribution of pyramidal (Zone 1 > Zone 2 > Zone 3) training and its focus on a high training volume in Zone 1, the design of the performed pyramidal and threshold training could be an explanation for this and should therefore be critically examined. In our study, we adapted the performed pyramidal training to given recommendations for cancer patients (Schmitz et al. 2010) and therefore performed only two training sessions per week to avoid potential overtraining. The significant within‐group improvements in endurance capacity confirm that this training stimulus was sufficient for our population. However, the mean training volume spent in Zone 1 (55%) was low compared to another study investigating pyramidal training (Filipas et al. 2022). This relative low time in Zone 1 could explain the lack of superior effects of the pyramidal training. Also, the intensity of threshold training in the present study (i.e., 97% of IAT) was much higher than the intensity of threshold training (i.e., PO between LT and IAT and 95% of PO at VT_1_) chosen by studies comparing different intensity distributions (Neal et al. 2013; Zapata‐Lamana et al. 2018). The higher intensity was chosen to allow for an isocaloric comparison of training methods without spending too much training time, considering recommendations for cancer patients (Campbell et al. 2019; Schmitz et al. 2010). The higher intensity of the performed threshold training may have resulted in a training stimulus inducing similar adaptions in aerobic metabolism (Hawley and Stepto 2001; Mijwel et al. 2018) as the pyramidal training.

The examination of our isocaloric training design indicates that it was appropriate, which is supported by the similar energy expenditure and increase in energy expenditure of both groups (Figure 2C,D). However, especially for pyramidal training, energy expenditure increased strongly from training week one to week two (9 ± 17 kJ/session). This large difference in terms of energy expenditure from week one and week two in the pyramidal training group can be explained by the lack of experience with the HIIT. It usually takes a while for unexperienced exercisers to adapt to HIIT and find an appropriate work rate for the high‐intensity intervals to reach 85%–95% HR_peak_. For this reason, we recommend that future studies in this area should include a familiarization period. Furthermore, the constant adjustment/increase of the individual work rate, which is scarce in clinical exercise interventions (Carvalho et al. 2021), could have ensured that the training stimulus was always adequate in both groups. No comparable studies are available in the current literature, so a direct comparison is not possible. However, two studies in breast and prostate cancer patients (Dolan et al. 2016; Martin et al. 2015), which also used a work‐matched training design, showed that high‐intensity and moderate‐intensity training led to comparable improvements in endurance capacity. Consequently, the aforementioned studies and our results indicate that the total energy expenditure is a major determinant of improvement in endurance capacity. This is underlined by a meta‐regression which was able to prove in the context of patients with chronic heart failure that total energy expenditure is a primary determinant for enhancing endurance capacity (Vromen et al. 2016). Clearly, the above indicates that a comparison of isocaloric training regimens is of enormous relevance for a concrete interpretation of training effects and should be considered more frequently in future analyses in the clinical setting.

Analysis of the load parameters showed that 93% of the participants met at least two criteria of maximal exhaustion at baseline and 89% at post‐intervention testing and that HR_peak_, RER_peak_, and RPE did not change from baseline to post‐intervention assessments. This indicates that the intensity targets obtained from baseline testing were appropriate (Schneider, Schlüter, Wiskemann, et al. 2020b). Furthermore, it indicates that missing differences in training adaptation between groups are not attributable to the fact that one group might have spent less effort in the CPET than the other. Interestingly, the percentage of participants who have met the verification criterion has increased in both groups. One reason for these results could be low familiarity with CPET and maximal exhaustion at baseline. In this case, it might be assumed that the pyramidal training group might have become more familiar with high exercise intensities than the ThT. This is confirmed by the fact that the percentage of participants who have met the verification criterion has increased by 11% in the pyramidal training group and 5% in ThT. Support for this point is also provided by the energy expenditure data, which show a sharp increase in the energy expenditure of the pyramidal group from the first to the second week, suggesting better familiarization.

Finally, the results show that a threshold training requires significantly less time than a pyramidal training (on average 30 vs. 38 min per training session) to achieve similar positive effects and could therefore be beneficial for patients who have less time. In contrast, pyramidal endurance training that includes high, moderate, and low‐intensity components might be beneficial for patients who prefer more variety in their training. So, threshold and pyramidal trainings appear to be safe and feasible for cancer patients after the completion of their therapy and offer new opportunities to provide adequate training to cancer survivors with different needs.

## 5 Conclusion

Based on this analysis, we conclude that breast and prostate cancer patients after the end of therapy were not able to achieve a polarized training intensity distribution. However, breast and prostate cancer survivors can perform both pyramidal and threshold endurance training. These two training regimens led to similar significant improvements in endurance capacity, with no evidence of superiority of either regimen. Moreover, pyramidal training required slightly longer training sessions on average, indicating that cancer survivors with limited time might prefer ThT, whereas cancer survivors who favor varied training sessions might prefer a pyramidal intensity distribution. In our study, the consideration of matched energy expenditure allowed an adequate comparison of the two exercise intensity distributions. However, further research might explore whether a true polarized training intensity distribution and other training regimens with higher training load are feasible and effective in cancer survivors.

## Conflicts of Interest

The authors declare no conflicts of interest.

## Supporting information

Supplementary Material

## Data Availability

The data that support the findings of this study are available from the corresponding author upon reasonable request.
