# Supplementary material for: Effects of Different Training Intensity Distributions on Endurance Capacity in Breast and Prostate Cancer Survivors: A Randomized Controlled Trial
Source: Eur J Sport Sci. 2025 Apr 8;25(5):e12287. doi: 10.1002/ejsc.12287 (PMC11977512; doi:10.1002/ejsc.12287)
Supplement: Supplementary file 1 — Supplementary Material [file EJSC-25-e12287-s001.docx]

**Supplementary Material**

**Supplementary Table 1: Planned and performed training intensity distribution of training groups.**

|  | **Training** | |  |
| --- | --- | --- | --- |
|  | **ThT** | **POL** | ***p*** |
| **Planned** | | |  |
| HR – Time in Zone 1/Zone 2/Zone 3 | 0/100/0 | 79/0/21 |  |
| Polarization-Index [a.U.] | n.a. | > 2.00 |  |
|  |  |  |  |
| **Performed** | | |  |
| HR – Time in Zone 1 [%] (mean ± SD) | 4 ± 8 | 55 ± 15 | < .001 |
| HR – Time in Zone 2 [%] (mean ± SD) | 59 ± 38 | 34 ± 18 | 0.003 |
| HR – Time in Zone 3 [%] (mean ± SD) | 37 ± 39 | 11 ± 7 | 0.001 |
| Polarization-Index [a.U.] (mean ± SD) | n.a. | 1.2 ± 0.7 | n.a. |
| *Significant for p < .05 | | | |

**Supplementary Table 2: Criteria for maximal exhaustion.**

|  | **Pyramidal training group**  **(planned POL)**  (mean ± SD) | |  | **Threshold training group**  (mean ± SD) | |  | **ANOVA *p*** | | |
| --- | --- | --- | --- | --- | --- | --- | --- | --- | --- |
|  | **PRE** | **POST** |  | **PRE** | **POST** |  | **Time (η²)** | **Group (η²)** | **Group** x **Time (η²)** |
| HR_peak_ | 154 ± 14 | 157 ± 15 |  | 153 ± 14 | 154 ± 17 |  | 0.19 (0.003) | 0.57 (0.006) | 0.32 (0.002) |
| RER_peak_ | 1.16 ± 0.08 | 1.20 ± 0.09 |  | 1.15 ± 0.06 | 1.16 ± 0.09 |  | 0.17 (0.01) | 0.22 (0.02) | 0.19 (0.01) |
| bLA_peak_ | 7.92 ± 1.83 | 8.54 ± 2.37 |  | 7.36 ± 1.80 | 7.67 ± 2.35 |  | 0.03* (0.01) | 0.18 (0.03) | 0.47 (0.001) |
| RPE | 18 ± 2 | 18 ± 1 |  | 19 ± 1 | 18 ± 1 |  | 0.51 (0.002) | 0.51 (0.006) | 0.21 (0.008) |
| *Significant for p < .05 | | | | | | | | | |

**Supplementary Figure 1:** **Individual changes in VO_2peak_ over the intervention period.**

**
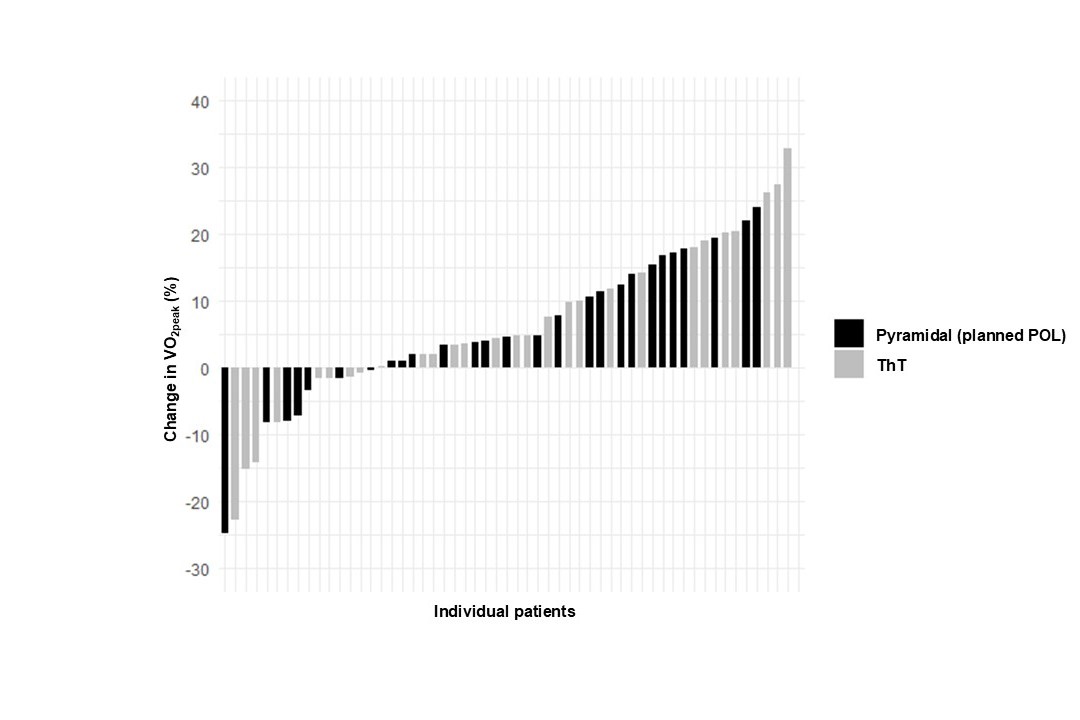
**
